# Co-occurring psychotic and eating disorders in England: findings from the 2014 Adult Psychiatric Morbidity Survey

**DOI:** 10.1186/s40337-022-00664-0

**Published:** 2022-10-18

**Authors:** Ellen Rodgers, Steven Marwaha, Clara Humpston

**Affiliations:** 1 Institute for Mental Health, School of Psychology, University of Birmingham, B15 2TT Birmingham, UK; 2 Birmingham and Solihull Mental Health Foundation Trust, B1 3RB Birmingham, UK; 3 Department of Psychology, University of York, YO10 5DD York, UK

**Keywords:** Eating disorders, Psychotic disorders, Comorbidity, Mood instability, Epidemiology, Household survey

## Abstract

**Background:**

Psychotic disorders and eating disorders are complex mental illnesses associated with increased mortality and functional impairment. This study aimed to investigate the co-occurrence and relationships between eating disorders and psychotic disorders and assess the mediation effect of mood instability.

**Methods:**

This study used data from the Adult Psychiatric Morbidity Survey (APMS) 2014, a general population-based survey in England. Participants (total *N* = 7546, female *N* = 4488, male *N* = 3058, mean age = 52.3 years) were categorised based on psychotic disorder status into the groups of probable psychosis, diagnosed psychosis, and healthy controls without psychosis. The dependent variable of this study was the presence or absence of an eating disorder, with mood instability as the mediator. Logistic regression and mediation analyses were conducted to assess the relationships between these variables.

**Results:**

Both probable and diagnosed psychoses were significantly related to the presence of an eating disorder, and mood instability was found to be a mediating variable with moderate effect.

**Conclusion:**

The present study demonstrates a significant relationship between eating disorders and psychotic disorders in the English general population, indicating higher levels of co-occurrence between these two groups of disorders than when compared with healthy controls. The findings also suggest the relationship between eating and psychotic disorders is mediated, to an extent, by the presence of mood instability traits. Future research could extend the present study’s findings through assessing whether specific eating disorders are more significantly related to psychotic disorders than others.

## Plain English summary

Eating disorders as well as psychotic disorders pose a significant risk to those diagnosed, with anorexia nervosa having the highest mortality rate among all mental disorders. The two groups of disorders are shown to be related as those with psychotic disorders are more likely to also be diagnosed with an eating disorder. Further, the relationship between eating and psychotic disorders could be due to trait mood instability. Mood instability is defined as the rapid switching between moods that those affected cannot control easily and has been found in individuals who are diagnosed with either an eating or a psychotic disorder. This leads to the question whether mood instability is involved in the relationship between psychotic and eating disorders. As individuals with both eating and psychotic disorders experience high levels of distress and treatment is challenging, it is important to understand the nature of the relationship between these two disorders.

This study used data collected from a population-based survey in England to examine whether psychotic disorders were related to eating disorders, and whether mood instability was implicated in the relationship. The study found that psychotic and eating disorders were significantly related, and mood instability had a moderate effect on the relationship.

## Background

Psychotic disorders (PD) and eating disorders (ED) are complex mental illnesses associated with increased mortality [1, 2] and poorer quality of life [3, 4]. EDs have become increasingly common over the past decade; in addition to the negative impact on the individual, anorexia nervosa holds the highest mortality rate across all psychiatric disorders [2]. Moreover, ED diagnosis is challenging due to the complexity of the disorders, which results in challenges with treatments too [2, 5].

The relationship between PD and ED remains unclear and not systematically well-studied [6, 7], although some evidence suggests the two groups of disorders are related to an extent [8, 9]. Much of the evidence supporting the link between ED and PD comes from case studies. Hugo and Lacey [8] report four cases of patients with co-occurring PD and anorexia nervosa (AN)/bulimia nervosa (BN). In two patients, psychotic symptoms worsened following improvement in ED symptoms, leading the authors to conclude that in these patients, disordered eating behaviours protected against psychotic symptoms [8]. Seeman [7] supports this, explaining that disordered eating offers psychological control. The control one may gain from restricting and changing eating behaviour is said to compensate for otherwise low self-efficacy in individuals at risk of psychosis, hence it is possible to extend Seeman’s [7] hypothesis surrounding control and disordered eating to those with a PD. Researchers describe reduced feelings of agency in psychosis as ‘passivity symptoms’ [10] as those affected have issues with feelings and judgements of agency regarding their actions [11]. Hauser et al. [12] support this, stating that individuals who experience reduced cognitive and emotional control via psychosis use disordered eating to regain control. Consequently, PD and ED may be linked through the role of cognitive control.

In early accounts of EDs, the patients’ disordered thinking styles were regarded as psychotic [13]. This idea is supported by recent empirical studies. In Miotto and colleagues’ study, they assessed the level of psychotic symptoms in ED patients (with BN or AN) and compared them to a control group. ED patients reported more frequent experiences of psychotic, including paranoid, symptoms [9]. Similar experiences with psychotic symptoms have been reported in binge eating disorder (BED) patients. Aragona and colleagues found that increased psychotic experiences were linked with the severity of BED symptoms. Whilst findings from Aragona et al. [13] should be interpreted with caution due to the lack of a control group, other researchers support the existence of delusional thinking in EDs too. Behar, Arancibia, Gaete, Silva & Meza-Concha [14] found that individuals diagnosed with EDs showed delusional thinking patterns like those observed in PD. Delusions in EDs focus on body image and weight, suggesting that PD and ED may be linked through the shared symptom of delusional or delusion-like ideation [13, 14].

Another factor that may link the psychopathology of PD and ED is mood instability. Mood instability is defined as rapid oscillations in mood where individuals struggle to regulate their mood or the consequences associated with their behaviour [15]. Unstable mood is observed in many mental disorders, including PD [16] and ED [17]. As mood instability is associated with increased service use and suicidal ideation [18], it is important to consider the role unstable mood may have in co-occurring PD and ED.

At present, the evidence surrounding the role mood instability has in the relationship between PDs and EDs is sparse. A limited body of research suggests that those with AN or BN may be susceptible to psychotic symptoms and unstable mood [17]. As mood instability has been implicated in PD and ED individually [16, 19, 20], there is a possibility that the relationship between PD and ED may also be mediated by mood instability.

It is often thought that psychotic symptoms result from cognitive disturbances [21]. Cognitive distortions that lead to these symptoms are largely due to a breakdown in normal cognitive function, including the observation that individuals with PD often characterise self-generated cognitions as unfamiliar or coming from an external source [21]. From this, it is possible that the explanation for errors in source monitoring observed in PD are due to other, wider impairments in cognition. Fusar-Poli et al.’s [22] meta-analysis supports this, as individuals diagnosed with PD showed poorer executive function skills. This is extended by Aase et al. [23] who found that poor executive functioning resulted in increased positive symptoms of psychosis (such as hallucinations and delusions). Consequently, atypical cognitive function appears to have a role in PD [21–23]. As mentioned previously, low self-efficacy and cognitive control are observed in those with co-occurring PD and EDs [7]. Therefore, cognitive disturbances and poor cognitive control may also explain why PD and ED may be related.

Based on previous research highlighting the potentially significant relationship between the groups of PDs and EDs [8, 9, 13] and the possible role of mood instability in this relationship [17], the present study aimed to answer the following research questions:

1. Is the presence of psychotic symptoms, such as paranoid and hallucinatory experiences, associated with the presence of a diagnosed eating disorder?
2. Does the strength of association between psychotic symptoms and diagnosed eating disorder differ between groups of individuals who have diagnosed or probable psychosis, as compared with controls?
3. Does mood instability mediate the relationship between psychotic symptoms and diagnosed eating disorders?

## Methods

### Data collection and participants

The data used in this study were collected as part of the Adult Psychiatric Morbidity Survey (APMS) 2014. To select participants, the APMS 2014 employed a random, multi-stage stratified sampling method based on postcode sectors in England (of which there were 2550), ensuring an even spread across England. This was followed by another round of random sampling of privately owned household addresses within the sectors. From the selected households, one adult over the age of 16 was randomly selected for an interview resulting in 7546 participants (female *N* = 4488, male *N* = 3058, mean age = 52.3 years) with responses to over 1000 variables in the final dataset. Participants were able to decline the invitation to participate as participation was voluntary.

Following participant consent, interviewers contacted participants for a semi-structured interview where multiple screens for psychiatric disorders were administered. The APMS 2014 used screening tools to assess common mental disorders, mental health treatment and service use, posttraumatic stress disorder, psychotic disorder, autism spectrum disorder, personality disorder, attention-deficit/hyperactivity disorder, bipolar disorder, alcohol dependence, suicidal thoughts, suicide attempts and self-harm. Depending on a participant’s response determined whether they were contacted for an additional, second semi-structured interview. The APMS 2014 was designed to be representative of the entire population in England, hence the extensive sampling method and large sample size.

A full, detailed breakdown of the APMS 2014 can be found in McManus, Bebbington, Jenkins & Brugha [24].

### Psychotic symptom assessment

The Psychosis Screening Questionnaire (PSQ; [25]) was administered to participants during the first phase of APMS 2014 interviews. Participants were asked several questions used to indicate the presence of probable psychosis or a diagnosed PD. This also included asking participants whether they took antipsychotic medication, if they had been hospitalised for mental or emotional problems, whether they had a PD diagnosis, as well as a positive response to the PSQ items concerning experiences of auditory hallucinations and persecutory delusions.

The PSQ is comprised of five sections assessing different elements associated with psychotic experiences and PD: hypomanic mood, thought interference, paranoia, strange experiences, and auditory hallucinations. Participants gave yes/no/unsure answers to the questions, based on their experiences in the past year. The authors, Bebbington and Nayani, purposely made the first questions of each section broad so as not to overwhelm participants and to exclude sections if negative responses were given. The number of individuals who complete the PSQ and are identified as having potential PD is similar to that of those who would be clinically diagnosed using a standardised measure [26, 27], suggesting that the PSQ has good construct validity. From their original study, the PSQ also possesses very high levels of specificity (98 − 100%) for use as a screening instrument.

Following the completion of the PSQ within the first phase, those who were identified as having probable psychosis or a PD were invited to participate in phase two, a second semi-structured interview. This equated to 6% of the original population. The Schedules for Clinical Assessment in Neuropsychiatry [28] was administered by a trained clinician. This process is reported in full by McManus et al. [24].

In the present study, individuals who answered yes to the PD diagnosis in the past 12 months by a medical professional from the variable (PDg12Doc_ReB) which in turn formed the diagnosed psychosis (clinical PD) group. This variable was chosen as it asks participants directly if they have been diagnosed with a PD or schizophrenia in the past 12 months by a medical professional. Those whoscreened positive in the probable PD (PsychDisProb) variable formed the probable PD group. Those who answered yes to this variable were identified as positive for psychotic symptoms following the SCAN but were not identified as having a formal diagnosis for PD or schizophrenia. All other individuals formed the control group.

### ED assessment

The presence of an ED was indicated by responses to several questions on the APMS 2014. Participants were categorised as having an ED if they self-identified as having an ED when asked by the interviewer, had an ED diagnosis from a medical professional, or were receiving treatment for an ED at the time of the interview. The APMS 2014 also includes information on the age of onset of ED but does not include a specific questionnaire or screen to examine ED symptoms.

Both grouping variables for the clinical PD and probable PD groups were based on participant experiences in the past 12 months. Thus, for consistency, the present study uses the variable from the APMS 2014 dataset which asks participants whether they were diagnosed with an ED in the past 12 months by a medical professional (PDg9Ly_ReB). Although it is known that EDs include many varied disorders that are categorised by different symptoms, the present study is not able to differentiate between the different EDs and therefore cannot assess the extent to which the specific ED disorders are related to PD presence. This is due to the way the ED data was collected in the APMS. Thus, there will be just two levels to the binary ED variable: positive for diagnosed ED, or negative for diagnosed ED.

### Mood instability

Mood instability was defined using the personality disorder scale, as administered during the APMS 2014. Participants who endorsed either of the PD79 or PD82 items were categorised as having unstable mood. The personality disorder scale is primarily designed to measure the presence of a personality disorder, yet the items selected for analysis in the present study are also associated with mood instability. PD79 asks if participants believe they are ‘often doing things impulsively’, whilst PD82 asks if participants feel they ‘often have a lot of sudden mood changes’. These were both dichotomous variables with ‘yes’ or ‘no’ answers.

### Data analysis

Data analysis was conducted using the programming language R in R studio and STATA SE/16. Analyses were weighted using the ‘weight-core’ variable in the APMS 2014 dataset. This accounts for non-response across the data to ensure results are representative of the general population. This also reduces the risk of over or underestimating the true prevalence of the disorders assessed in the APMS 2014. Any incomplete data due to withdrawal from the study or non-response was excluded from the final analysis.

Descriptive statistics were obtained based on sociodemographic features such as age, gender, ethnicity, socioeconomic status, and employment status for the clinical (diagnosed) PD, probable PD and control groups, with weighted Chi-squared tests carried out between diagnosed PD and probable PD groups accordingly.

We included three logistic regression models in the analyses: one assessing the relationship between probable PD and ED presence, one assessing the relationship between diagnosed PD and ED presence, and an alternative model including specific psychotic symptoms (delusions and hallucinations). Regression analyses were conducted in two stages, with sociodemographic variables that demonstrated significant differences in Chi-squared tests entered into stage 1 as explanatory variables, and various PD variables of interest were entered into stage 2 of the model as additional explanatory variables. ED presence was the dependent variable in all analyses. Weighted logistic regression analyses were performed to obtain odds ratios and 95% confidence intervals for ease of interpretation.

Lastly, mediation analyses assessed the effect the two mood instability variables (PD79 and PD82) had on the association between PD groups and ED presence. This measured the extent to which impulsive behaviour and unstable mood impacted the relationship between diagnosed PD, probable psychosis and ED. The KHB command in STATA was used to conduct this mediation analysis. This method of mediation analysis compares logistic regression models using the average partial effect [29].

## Results

Descriptive statistics can be found in Table 1. The prevalence of diagnosed PD as diagnosed by a medical professional in the past year in the APMS 2014 survey was 0.6% (*N* = 45), whilst the prevalence of probable PD was 1.2% (*N* = 94).

**Table 1:** Descriptive statistics of the sample with results from weighted Chi-squared tests

	Diagnosed Psychosis (N = 45)			Probable Psychosis (N = 94)		
	N (% of total)	Weighted χ^2^	*p*	N (% of total)	Weighted χ^2^	*p*
**Gender**		6.29	0.01		0.049	0.83
Male Female	27 (60) 18 (40)			39 (41.5) 55 (55.5)		
**Employment**		30.46	< 0.001		75.48	< 0.001
Employed Unemployed (including not economically active)	5 (11.1) 40 (88.9)			22 (10.6) 72 (89.4)		
**Ethnicity**		2.18	0.14		0.14	0.71
White Non-white	38 (84.4) 7 (15.6)			84 (89.4) 10 (10.6)		
**Marital Status**		11.88	< 0.001		27.05	< 0.001
With others Alone	10 (22.2) 35 (77.8)			22 (10.6) 72 (89.4)		
**Age band**		18.10	0.006		11.31	0.08
75+ 65–74 55–64 45–54 35–44 25–34 16–24	2 (4.4) 5 (11.1) 9 (20.0) 6 (13.3) 14 (31.1) 8 (17.8) 1 (2.2)			3 (3.2) 9 (5.7) 17 (18.1) 21 (22.3) 21 (22.3) 16 (17.0) 7 (7.4)		

In the diagnosed PD group, there was a higher proportion of males (60%), unemployed individuals (89%), people of white ethnicity (84%), and those living alone (78%). The greatest proportion of PD diagnoses was observed in the 35–44 age group (31%).

In the probable PD group, there were a higher proportion of females (59%), individuals in the 35–44 and 45–54 age groups (45%), those who were unemployed (89%), people of white ethnicity (89%), and those living alone (77%).

The first regression analysis revealed that ED presence in the past year was significantly associated with the co-occurrence of diagnosed PD (p < 0.001, odds ratio (OR):12.92, 95% Confidence Interval (CI):3.36–49.70). In this model, age was found to be statistically significant (p = 0.013, OR:0.84, CI:0.73–0.96), as was gender (p = 0.023, OR:2.4, CI:1.14–5.08), and marital status (p < 0.001, OR:0.25, CI:0.11–0.55).

The second logistic regression revealed that ED presence in the past year was statistically significantly associated with probable PD (p < 0.001, OR: 28.42, CI:11.42–70.73). In this model, only marital status was found to be significant (p = 0.0011, OR:0.24, CI:0.10–0.57).

Due to the highly significant results from the two models, a third regression (‘Alt model’) was performed using the PSQ3a (persecutory delusions: felt that people were deliberately acting to harm you/your interests) and PSQ5a (auditory hallucinations: heard voices saying quite a few words or sentences) from the PSQ. These items aim to assess whether individuals have experienced clinically meaningful delusions or hallucinations typical of a PD, meaning that they would have also already endorsed PSQ3 and PSQ5 which ask about less specific psychotic-like experiences. However, the regression analyses performed for PSQ3a/PSQ5a and ED diagnosis did not produce statistically significant results (see Table 2).

**Table 2:** Results from weighted logistic regression with eating disorder diagnosis as outcome (dependent) variable. OR, odds ratios; CI, confidence intervals

Measure	*β* (95% CI)	*P*	OR (95% CI)
**Model 1**			
*Stage 1*			
Age Gender Ethnicity Marital Status	-0.18 (-0.31–0.038) 0.88 (0.13–1.63) -0.66 (-1.41–0.084) -1.37 (-2.17 – -0.58)	0.012 0.022 0.081 < 0.001	0.83 (0.73–0.96) 2.40 (1.14–5.08) 0.51 (0.24–1.09) 0.25 (0.11–0.56)
*Stage 2*			
Diagnosed Psychosis	2.56 (1.21–3.91)	< 0.001	12.92 (3.36–49.70)
**Model 2**			
*Stage 1*			
Ethnicity Marital Status	-0.13 (-0.99–0.72) -1.42 (-2.27 – -0.57)	0.77 0.0011	0.88 (0.37–2.09) 0.24 (0.10–0.57)
*Stage 2*			
Probable Psychosis	3.35 (2.44–4.26)	< 0.001	28.42 (11.42–70.73)
**Alt Model**			
*Stage 1*			
Ethnicity Marital Status	-2.04 (-3.79–0.96) 1.30 (-3.10–0.49)	0.25 0.16	0.24 (0.023–2.61) 0.27 (0.045–1.64)
*Stage 2*			
Persecutory Delusions Auditory Hallucinations	-0.32 (-2.12–1.48) 0.20 (-1.82–2.21)	0.73 0.85	0.73 (0.12–4.39) 1.22 (0.16–9.12)

Mediation analyses were conducted to assess the role mood instability had in the relationship between ED and diagnosed/probable PD. Both mood instability variables combined mediated the effect of diagnosed PD and ED by 18.31%. The variable ‘often doing things impulsively’ accounted for 8.22% of the effect diagnosed PD had on ED presence. Yet, the variable ‘often have a lot of sudden mood changes’ accounted for 15.36% of the effect diagnosed PD had on ED presence.

10.7% of the relationship between probable PD and ED was mediated by both mood instability variables. 5.69% of the effect probable PD had on ED presence was accounted for by the variable ‘often doing things impulsively’, whereas 8.90% of the effect probable PD had on ED presence was accounted for by the variable ‘often have a lot of sudden mood changes.’ For total, direct, and indirect effects of the mediator variables, see Table 3.

**Table 3:** Results from mediation analyses with eating disorder diagnosis as dependent variable. OR, odds ratios; CI, confidence intervals. Asterisks indicate levels of statistical significance

	Probable Psychosis	Diagnosed Psychosis
OR (95% CI), p*** (< 0.001), p**(< 0.01), p*(< 0.05)		
**Impulsivity + Mood Instability**	10.70%	18.31%
Total Direct Indirect	12.86 (6.45–25.65) *** 9.79 (4.68–20.46) *** 1.31 (1.04–1.66) **	6.23 (1.78–21.82) ** 4.46 (1.25–15.95) ** 1.40 (1.08–1.82) **
**Impulsivity**	5.69%	8.22%
Total Direct Indirect	13.26 (6.75–26.06) *** 11.45 (5.80–22.58) *** 1.16 (1.02–1.31) **	6.70 (1.94–23.15) ** 5.73 (1.66–19.84) ** 1.17 (1.00–1.37)
**Mood Instability**	8.90%	15.36%
Total Direct Indirect	13.03 (6.47–26.2) *** 10.37 (4.94–21.78) *** 1.26 (1.01–1.31) *	6.46 (1.89–22.08) ** 4.85 (1.39–16.87) ** 1.33 (1.07–1.66) **

## Discussion

From the above findings, we can tentatively suggest that there is a significant relationship between diagnosed PD and a diagnosis of an ED, and probable PD and a diagnosis of an ED. Such that, individuals with a diagnosed PD, or those in the probable PD group, are more likely to have also been diagnosed with an ED compared to controls. Mood instability was found to account for a small to moderate amount of the association between diagnosed PD and ED, with emotional instability as opposed to behavioural impulsivity being the strongest mediator variable. Mood instability was found to have a smaller mediation effect in the relationship between probable PD and ED, but emotional instability was the strongest mediator in this model too. Sociodemographic information, including age, gender, employment status and marital status, also demonstrated statistically significant differences between the PD groups; however, even after accounting for these sociodemographic variables in the regression models, the relationship between the PD groups and an ED diagnosis persisted.

When discussing the symptoms and experiences of individuals diagnosed with ED, researchers highlight similarities with PD. Those with AN are described as having delusional thinking patterns surrounding their body image and weight [14, 30]. Behar et al., [14] state that delusional beliefs in EDs exist on a spectrum from over-valuing body image to overt delusion surrounding one’s body and size [30]. These delusions in EDs, it is said, encompass the denial of one’s own diagnosis [14]. Denial or lack of insight, along with the finding that weight loss can exacerbate psychotic symptoms, such as hallucinations and delusions, [8, 14] suggest that there is potentially a psychotic element to EDs, supporting the idea that PD and ED are related.

Mood instability has been found to impact ED development and symptoms as well. Brown, Hochman and Micali [20] investigated the role mood instability during childhood played in ED development. Emotional instability and depressed mood were both found to be significant predictors of disordered eating behaviour at age 14 and 16. This is a particularly important finding surrounding the influence mood instability has on ED development. Low mood and depressive symptoms have been linked to the occurrence of binge/purge behaviour in BN patients [31] as well as heightened levels of impulsive behaviour [32]. Thus, it could be argued that unstable mood, particularly unstable negative moods, are significantly associated with EDs [20, 31, 32].

Further research from Miniati et al., [17] suggests that mood instability is found in patients with AN or BN. Additionally, BN patients were more prone to psychotic symptoms and depressive moods compared to AN patients. Therefore, these findings could be interpreted as linking psychosis, ED, and mood instability. This, along with the fact that mood instability increases the risk for suicide in both PD and ED patients [17, 19] mean that it is important to further investigate the role mood instability plays in the relationship between ED and PD.

A possible explanation for the findings in the present study may be genetics. It has been shown that respectively, EDs and PDs are moderately heritable disorders [33, 34], that may be related at a genetic level [35]. Findings from Zhang et al. [35] suggest that in families where an individual has a diagnosed ED, their relatives have a greater risk of being diagnosed with schizophrenia than control families. It has also been found that schizophrenia and mood instability have a shared genetic basis, which may help to explain the role mood instability had as a mediator between ED and PD in the present study [36]. Hindley et al. [36] emphasise the findings from their study should be interpreted with caution as the correlation between gene loci in mood instability and schizophrenia is weak to moderate. However, these findings could be seen to support the results observed in the present study when used alongside those by Zhang et al.: that EDs and PDs are significantly related and mood instability acts as a mediator, due to shared genes between the disorders which may go beyond EDs and PDs and extend into other mental health disorders and even physical health conditions. The co-occurrence of EDs and PDs could indeed be viewed through the lens of comorbid disorders beyond these categories, for example, the mediating role of mood instability may also at least partly explain the high rates of depression and anxiety associated with ED diagnoses. However, good quality evidence on comorbid PD and ED is scarce compared to data assessing mood disorders alongside EDs.

The finding that PD and ED are associated with one another is in line with previous research [8, 9, 13] and is valuable as the body of evidence is relatively small [6]. The APMS 2014 dataset used in this secondary analysis was large in comparison to sample sizes of other studies. This study involved over 7000 participants, therefore the findings from this study could be widely generalisable.

The present study involved the use of a large dataset (*N* = 7546), which may be one of the largest cohorts used to assess the relationship between PD and ED. This is a strength as one of the largest studies prior to the present study included 112 participants [9], and other studies are small case studies. Additionally, the method in which the APMS 2014 data was collected means the prevalence of co-occurring PD and ED is likely to be representative of the general population of England.

This study, to our knowledge, is one of the first to assess the role of mood instability in relation to co-occurring PD and ED. Previous research from Miniati et al., [17] seemed to suggest that individuals with ED were more susceptible to mood instability and psychotic symptoms. However, Miniati and colleagues’ participants were not formally diagnosed with a PD. Consequently, the present study extends Miniati et al.’s [17] findings to those with formal diagnoses of both ED and PD, as well as those with probable PD. These findings can also be seen to support research that highlights the significant role of mood instability in ED [20, 32] and PD [16].

Moreover, the items used in the present study to establish mood instability can be found in a scale used by the APMS 2014 to measure personality disorder. Whilst most of the personality disorder scale looks at state-level mood disruption, the mediator variables used in the present study focus on trait mood instability. A previous longitudinal study from Brown, Hochman & Micali [20] emphasises that trait emotional instability is associated with the development of ED. This too was found in Ghaderi & Scott [37] as individuals with ED scored lower on trait emotional stability compared to controls. As trait-based mood instability variables have been shown to be most applicable to ED, this can be seen as a strength of the present study.

This study, however, has several limitations. As the APMS 2014 collected data from individuals living in private households in England, this means that individuals living in sheltered accommodation, individuals hospitalised at the time of data collection or homeless populations would not have been included in this study. A meta-analysis conducted by Ayano, Tesfaw and Shumet [38] found that the prevalence of PDs in homeless people (21.21%) was much greater, compared to the general population (12.7%). Similar results were found when comparing the prevalence of ED diagnosis in populations of homeless versus housed US Veterans. It has been demonstrated that homelessness increases the probability of developing an ED by 59% [39]. This suggests that the present study may under-estimate the true prevalence of both PDs and EDs as the study’s population does not include populations where the disorders are more prevalent [38, 39].

Another limitation is that the number of individuals in each of the PD groups was relatively small, the association between diagnosed/probable PD and ED therefore may be inflated, meaning findings should be interpreted with caution. In the APMS 2014, the data containing information on ED diagnoses is based on answers given to yes or no questions, meaning the variable is binary. It is argued that ED should be considered as dimensional [40, 41], as other authors note that disordered eating behaviour ranges from occasional restrictive diets to full-blown EDs [40]. Additionally, compared to a categorical approach of ED as well PD, dimensional approaches enhance classification and are more sensitive to comorbid symptoms such as impulsivity [41]. Therefore, the categorical approach adopted by the APMS 2014 can be criticised for not being sensitive enough to the differing levels of ED severity [40, 41].

An issue with the present study is that all ED are grouped into one variable. As noted by the DSM-5, there are four main ED categories: AN, BN, BED, and other specified feeding and eating disorder [42]. The main categories of ED diagnoses can be split further into more specific diagnoses which all differ. For example, individuals with purgative BN display a more negative emotional profile compared to non-purgative BN and non-specific BN [43]. This is supported by Anderson et al., [44] who found that AN-binge-purge patients and BN patients reported more difficulties with emotional regulation. Due to the differences observed across the ED diagnoses, it is possible to argue the grouping of ED patients into one group is not entirely appropriate. Consequently, the APMS 2014 is not able to distinguish what specific ED are more significantly associated with which PDs.

The same can be said for the PD variable used in the present study as there is no way to distinguish the different diagnoses from one another. The PDs, including schizophrenia, psychotic depression, and bipolar disorder, all have interrelated yet different cognitive profiles. Research suggests that the degree of cognitive impairment is worst in schizophrenia [45, 46]. Differences between PDs are also observed at a neurobiological level, as reported by Culbreth, Foti, Barch, Hajcak & Kotiv [47]. It was found that those with an affective psychosis showed the greatest brain activity in response to emotional stimuli. From this, it can be argued there are distinct differences between the PDs [45, 47] which are not detected in the present study. Therefore, it cannot be said whether a particular PD is more frequently observed in those with ED, due to the way participants were categorised.

Lastly, and perhaps the most important limitation of the present study, is the fact that the categorisation of the participants into groups (positive for ED, negative for ED, clinical PD, probable PD and controls) was reliant on self-report. Whilst the study makes use of the variables obtained by the APMS where participants were asked to declare if they were diagnosed with an ED or PD in the past 12 months by a medical professional, it is possible some responses may have been untrue. Particularly in ED it has been shown that patients often lie about the severity of their disordered eating, preferring to minimise the severity of their symptoms [48]. Therefore, the reliance on self-reported diagnoses in the present study may have resulted in potentially inaccurate estimates of the prevalence of PD, ED and ultimately the extent to which they are related. Hence the findings from the present study should be interpreted cautiously due to this limitation.

To address the limitations of the present study, future research could administer an ED questionnaire to specify which EDs are the most significantly associated with psychotic symptoms. Possible questionnaires are the Eating Disorder Inventory (EDI; [49]), the Eating Disorder Examination Questionnaire (EDE-Q; [50]) and the Eating Attitudes Test (EAT; [51]). The EAT is perhaps one of the most widely used psychometric tools for assessing EDs and has even been shortened to the 16-item version [51]. Despite its widespread use, the EAT is thought of as AN-specific [52], meaning the EDI may be best suited to comparing a range of EDs of differing severities. The EDI has been identified as having good psychometric properties, including good internal consistency [53, 54]. The EDE-Q has been shown to produce similar findings to that of the Eating Disorder Examination (EDE; [50]), the investigator-based interview the EDE-Q is based on. The EDE-Q has been shown to have good internal consistency and other psychometric properties [55]. Yet, a short version known as the EDE-Q7 [56, 57] is described as offering more robust psychometric properties than the original version [58].

Additionally, future research could aim to assess what specific PD are associated with ED, whilst also accounting for PD severity as seen in the present study. The SCAN [28] is a widely used method for assessing PD and can be used to specify which PD an individual has [59]. Although the SCAN was used in the present study, the APMS 2014 did not specify the prevalence of specific PD. Future research could also use the SCAN, but group individuals based on their specific PD and severity of their symptoms, thus allowing for comparisons to be made between different PD regarding the strength of their relationships with ED. This would develop a better understanding into the potentially psychotic elements in at least some ED [13, 14] which would in turn, improve treatment options for those individuals too.

Lastly, mood instability and its role in the relationship between PD and ED should be investigated further as this mediator variable is not well studied in this area of research. Although findings from Miniati et al., [17] and the present study suggest mood instability does mediate the relationship between PD and ED at least to a certain degree, further studies are clearly warranted including those adopting a longitudinal design.

## Conclusion

Overall, the present study supports previous research that PD and ED do co-occur in the general population. Despite the present study’s limitations, its findings contribute additionally to the relatively small area of literature. Our findings also suggest that mood instability has a moderately mediating role in the relationships between the two disorders.

## Data Availability

Data from the APMS can be officially obtained from the UK’s NHS Digital upon reasonable request.

## Abbreviations

APMS: Adult Psychiatric Morbidity Survey
EAT: Eating Attitudes Test
ED: Eating Disorder
EDI: Eating Disorder Inventory
EDE-Q: Eating Disorder Examination Questionnaire
PD: Psychotic Disorder
SCAN: Schedules for Clinical Assessment in Neuropsychiatry
